# Analysis of Sampling Methodologies for Noise Pollution Assessment and the Impact on the Population

**DOI:** 10.3390/ijerph13050490

**Published:** 2016-05-11

**Authors:** Guillermo Rey Gozalo, Juan Miguel Barrigón Morillas

**Affiliations:** 1 Facultad de Ciencias de la Salud, Universidad Autónoma de Chile, 5 Poniente 1670, Talca 3460000, Chile; 2 Departamento de Física Aplicada, Escuela Politécnica, Universidad de Extremadura, Avda. de la Universidad s/n, Cáceres 10003, Spain; barrigon@unex.es

**Keywords:** noise pollution, sampling methods, noise predictive capacity, noise annoyance

## Abstract

Today, noise pollution is an increasing environmental stressor. Noise maps are recognised as the main tool for assessing and managing environmental noise, but their accuracy largely depends on the sampling method used. The sampling methods most commonly used by different researchers (grid, legislative road types and categorisation methods) were analysed and compared using the city of Talca (Chile) as a test case. The results show that the stratification of sound values in road categories has a significantly lower prediction error and a higher capacity for discrimination and prediction than in the legislative road types used by the Ministry of Transport and Telecommunications in Chile. Also, the use of one or another method implies significant differences in the assessment of population exposure to noise pollution. Thus, the selection of a suitable method for performing noise maps through measurements is essential to achieve an accurate assessment of the impact of noise pollution on the population.

## 1. Introduction

A recent publication by the World Health Organization points out that noise pollution, ranked second among a series of environmental stressors for their public health impact and, contrary to the trend for other environmental stressors which are declining, is actually increasing in Europe [1].

Noise is known to have auditory and non-auditory health impacts [2]. Environmental noise causes both psychological and physiological non-auditory health effects and the evidence for the non-auditory effects is growing [3]. Specifically, road traffic is considered to be the main source of community noise pollution. The most important non-auditory effects of traffic noise are annoyance and sleep disturbance [4,5,6,7]. Annoyance is a feeling of displeasure that can result in adverse emotions including irritability, stress, fear, and even depression [8,9,10,11,12]; it is associated with health-related quality of life [13,14,15].

Nighttime noise exposure directly influences sleep disturbance causing body motility, sleep stage changes, delayed sleep onset latency, and nocturnal awakenings [2,6,16]. Sleep disturbances can lead to serious long term health effects and there is increasing evidence from epidemiological studies that indicate long-term noise exposure leads to cardiovascular diseases, obesity or diabetes [17,18,19,20,21].

In considering the adverse effects of noise, the European Commission recognised community noise as an important environmental problem and adopted the European Noise Directive to assess and manage environmental noise [22]. The Directive focuses on noise mapping that aims to evaluate the number of people exposed to environmental noise. The precision of noise maps is essential to an appropriate identification of affected places and for planning suitable control measurements. In addition, a proper management of noise pollution can lead to benefits in reducing air pollutants because of the relation between them [23,24].

The European Noise Directive has not only been applied to European countries, but has also been used as a reference by non-European countries [25,26,27,28]. For example, in Chile, where this study was developed, over recent years the government has supported a number of projects initiated to gather knowledge about the acoustic situation in the cities [29]. As in other countries, different methods or strategies have been used for noise mapping, such as computation methods or studies carried out with “*in situ*” measurements. The use of an appropriate sampling method is important for the precision of noise maps, because even computation methods need to be validated and calibrated using “*in situ*” measurements [30,31].

Nowadays the sampling methods more commonly used in noise mapping are based on systematic random sampling using a regular grid or on the stratification of urban roads [32,33,34,35,36,37,38,39]. There are also studies that carry out a stratification of land use after selecting any of the previous sampling strategies [40,41].

The grid method is the only sampling method that is accepted in an international standard, ISO 1996-2, that represents a verified reference for the measurement of noise levels in urban environments [42]. The grid method is widely used in many scientific fields because its use guarantees the statistical principle of equal probability and, moreover, a uniform coverage of the area under study. However, the grid method has other drawbacks. The standard says that the source of these problems stems from the existence of a high sound level variability in cases of proximity to the noise sources or the existence of large physical obstacles.

The stratification of urban roads is an increasingly popular method [34,36]. It is based on the generally accepted assumption that road traffic is the most important source of noise in cities, and for most streets it can be considered the main cause of the spatial and temporal variability of that noise. The stratification of urban roads used by a great number of researchers is based on information from the relevant ministries of transport [27,37,38,39,40]. These organisations classify the roads according to their main function and especially according to their design features.

In this context, our research group has been working for some years on the development of a sampling method for “*in situ*” noise measurements. We term this method the categorisation method. On the basis of the concept of street functionality, each stratum defined by the categorisation method presents a sound level variability that is lower than the total sound spatial variability in a city. This has produced significant improvements in both the reduction of the number of sampling points and in the estimation of noise levels in unsampled streets. Its usefulness has mainly been studied in Spanish cities with a wide range of populations: from 2000 to 3,250,000 inhabitants [43,44,45]. However, the economic development and urban planning of Chilean cities are different from the European cities analysed with the categorisation method in previous studies. Overall, European cities have typically been developed from a medieval historic centre with a complex street structure. Nowadays, shopping centres and administration centres are located in the historic centre. Chilean cities have a grid street plan in which streets run at right angles to each other, forming a grid. Also, another important difference is the fact that Chilean cities classify their roads according to a legislative procedure, whereas no standard classification exists for the roads in Spanish cities. The applicability of both methods based on roads classification has never been previously compared. In view of the above, the following objectives have been set out in this study:

- Compare the applicability and predictive capacity of two sampling methods—the legislative road classification and the categorisation method—in the assessment of urban noise in a Chilean city.
- Compare both sampling methods in terms of the prediction of exposure levels and the percentage of people annoyed.

Achieving these objectives will facilitate better understanding of the suitability of different noise situation sampling methods in cities. Information about the percentage of the population exposed in a Chilean city will also be provided. Until now this information has not been available in the Chilean cities evaluated. According to the European Noise Directive, the knowledge of the percentage of the population exposed is required for establishing effective preventive and, if necessary, corrective measures.

## 2. Methods

This study was conducted in the city of Talca (Maule region, Chile). Talca has a population of about 200,000 inhabitants (the population increases during the academic year due to the influx of university students) and is the tenth largest city in the country. The highest percentage of the active population (approximately 55%) works in the service sector, followed by the industrial sector (approximately 36%). This city does not have a historic centre and a high percentage of buildings have only one floor. The mean annual temperature and rainfall are 13 °C and 750 mm, respectively.

Three sampling methods were analysed: the grid method [42], road types established by the Ministry of Transport and Telecommunications of Chile (MTT) [46], and the categorisation method [45]. In order to compare the uncertainties using a similar sampling time the same number of sampling points (52) was selected for each measurement method. The grid method was analysed because it is accepted in an international standard, but its applicability was not compared with the other sampling methods.

### 2.1. Grid Method

**Figure 1:**
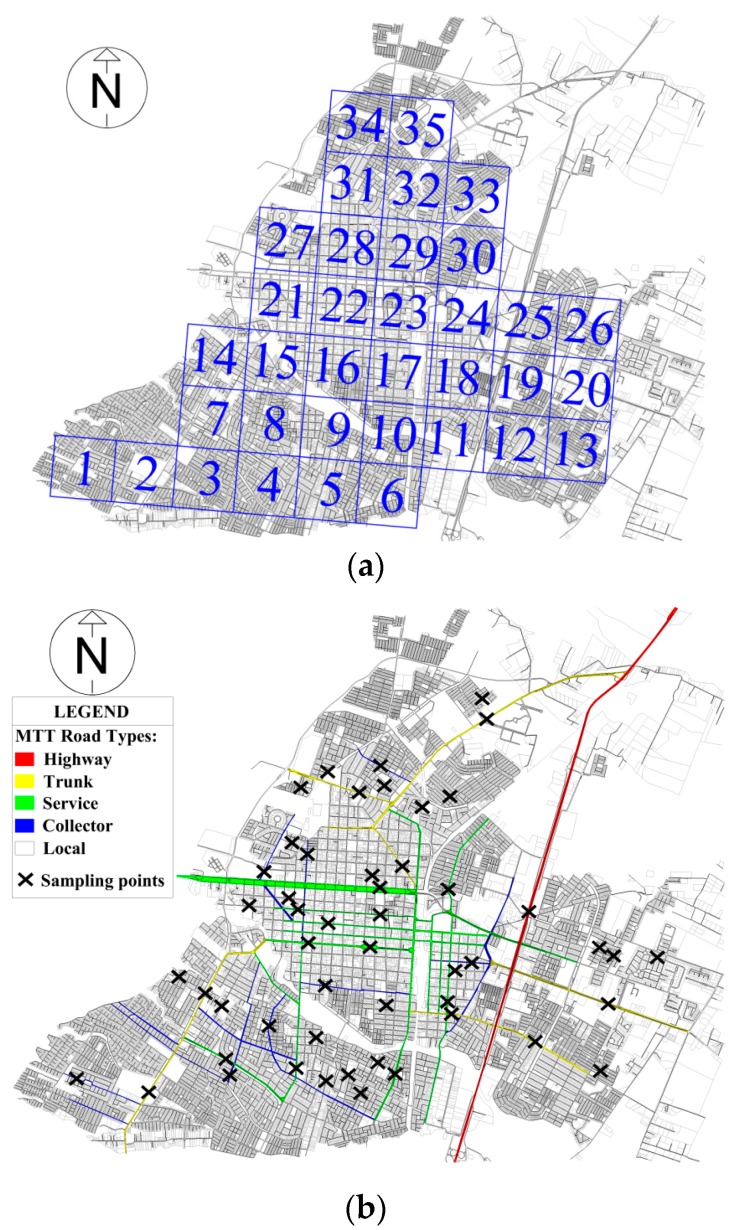

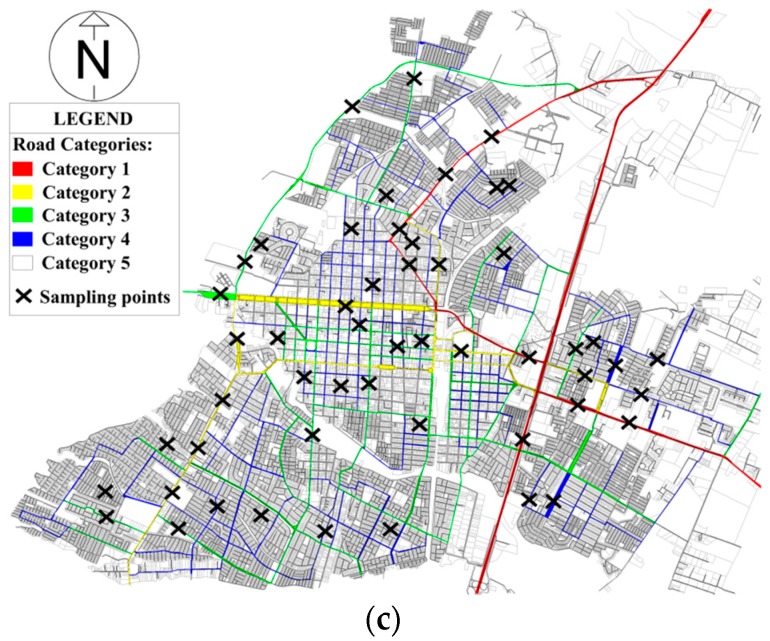
Sampling methods used in the city of Talca. (**a**) Sampling squares of grid method; (**b**) Ministry of Transport and Telecommunications (MTT) road types; (**c**) and categorisation method.

In the grid method, a grid is superimposed over a city map and the measurement points are located at the nodes of the square or at the nearest location when the nodes are inaccessible. The area of Talca is approximately 29 km^2^. A total of 35 squares with 52 sampling points were drawn on the city map using a grid square with 800 m of resolution. A similar square grid resolution has been used in previous studies [33]. Figure 1a shows the map of Talca with the grid used for this study.

### 2.2. Road Types Established by the MTT

The Ministry of Transport and Telecommunications of Chile (MTT) classifies urban roads according to their main function and their urban design features. However, in practice, urban characteristics, such as the width of the roads, are more relevant. Five types of roads are differentiated: highway, trunk, service, collector, and local. A similar classification has been used in recent acoustic assessment studies of cities in Chile and in other countries [27,37,38,39,40].

**Figure 2:**
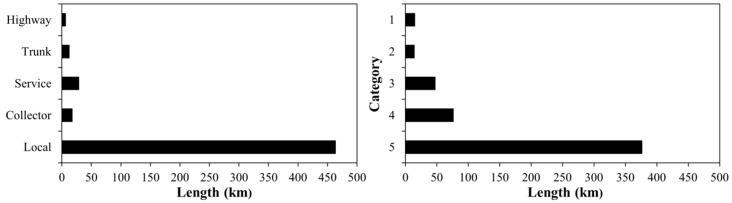
Length of road types and road categories in Talca.

The sampling points were then randomly selected along the total length of each road type taking into account two factors. First, in the types of roads with a greater length (see Figure 2), a greater number of sampling points were selected with a minimum of eight sampling points for each road type. Second, equivalent points (those points located on the same section of a street with no important intersection between them) were discarded. For this reason, only one sampling point was selected in the highway road type. Figure 1b shows the road types and locations of the sampling points: one point in highways, eight in trunk, twelve in service, eight in collector, and twenty-three in local road types.

### 2.3. Categorisation Method

As previously mentioned, the categorisation method is based on the concept of street functionality, that is to say, the functionality of the streets of the city as a communication path between different parts of the city and between the city and other urban areas. In addition, other variables such as the flow of vehicles, the type of traffic, the average speed, and urban variables may have a clear relationship with functionality [47]. The streets of Talca were classified according to the definitions proposed in the categorisation method established in previous work [48].

A strategy similar to the previous method was used to select the sampling points in each road category. Figure 1c shows the categorisation of different streets in the city and the locations of sampling points: eight points in Category 1, eight in Category 2, ten in Category 3, twelve in Category 4, and fourteen in Category 5.

### 2.4. Measurement Procedure

The measurements of different methods were carried out simultaneously from March to July 2015 following the ISO 1996-2 guidelines [42]. The measurements were performed on different working days and the sampling time for each measurement was 15 min. Previous studies [36,49] showed stability of the daily noise levels in the aforementioned months, and also these studies indicated that the main temporal variability of noise levels was among time-intervals within the day. At each sampling point, for each sampling strategy, at least five measurements were randomly selected in the following time-intervals: diurnal (from 07.00 to 19.00), evening (from 19.00 to 23.00), and nocturnal (from 23.00 to 07.00). A type-I sound level meter (2250 Brüel & Kjaer; Nærum, Denmark) was used with tripod and windshield and it was placed at a height of 1.5 m and at 2 m from the curb.

The A-weighted equivalent sound level (*L_Aeq_*) was used to analyse the results in the present study at different time-intervals of the day. The *L_Aeq_* registered in the diurnal period (from 07:00 to 19.00) and evening period (from 19.00 to 23.00) was very similar. For this reason, *L_Aeq_* from 7.00 to 23.00 (*L_d_*) was analysed. The noise descriptor *L_den_* was calculated following the guidelines of the European Noise Directive [22]. Other relevant information (traffic flow, types of vehicles, meteorological conditions, urban variables, *etc.*) was also noted.

### 2.5. Statistical Analysis

In the acoustic assessment in Talca, the applicability of different sampling methods was analysed using the calculated noise descriptors (*L_d_*, *L_n_* and *L_den_*) at each sampling point (*P_ij_*). The subscript “*i*” refers to the point code and the subscript “*j*” refers to the sampling method.

In the grid method there are no assumptions of the location of sampling points in urban roads. However, the location of the sampling points with respect to the traffic noise source was similar in the different sampling methods. For this reason, the sound values registered in the sampling points of the grid method were used to analyse the predictive capacity of the others two sampling methods. The noise value assigned to each square (*S_i_*) was the median value of the four nodes of the square. For each square, the interquartile range was calculated from these four values. Moreover, the difference in sound levels between adjacent grid points was calculated. This difference should not be greater than 5 dB according to ISO 1996-2 [42].

For the MTT road types and the categorisation method a similar statistic procedure was carried out. The value assigned to each road type (*R_i_*) or road category (*C_i_*) was the average of the sound levels measured at the sampling points (*P_ij_*). This value was the expected value for all of the other points located in the same road type or road category. The average sound value and its variability will determine whether the stratums formed by road categories or by road types present significant differences. This hypothesis was assessed using the nonparametric tests Kruskal-Wallis and Mann-Whitney *U* [50,51]. This hypothesis was not tested with an inferential analysis in previous studies that used a legislative road classification [27,37,38,39,40]. The Kruskal-Wallis test was used to compare all the road categories in order to identify any significant differences. When such differences were found, Mann-Whitney *U* tests were used to compare pairs of road categories. The Mann-Whitney *U* test evaluates whether two independent samples or observations come from the same distribution. To avoid any errors due to the use of data from the same population rather than randomly selected data, the Holm correction was used [52].

In contrast to previous statistical tests, the receiver operating characteristics analysis (*ROC*) was used to evaluate the discriminative capacity of the MTT road types and of categorisation method to differentiate the sound values of the sampling points between pairs of strata (stratum *i versus* stratum *j*) [45]. For the categorisation method and for MTT, the strata are the road categories and road types, respectively. The *ROC* analysis allows us to establish the upper and lower limits of the sound levels assigned to each stratum, to calculate the sensitivity (capacity to include previously assigned sampling points in the stratum), the non-specificity (proportion of sampling points that were not initially assigned to a certain stratum but that the *ROC* analysis indicated belonged to that stratum), and the predictive values (proportion of the sampling points that the *ROC* analysis assigned to a stratum that matched the strata to which they were initially assigned, relative to the total number of sampling points that the *ROC* analysis determined for the stratum). To do so, the following equations were used:

(1) $$sensitivity=\frac{n^{{}^{\circ}}\;of\;sampling\;points\;assigned\;correctly\;to\;stratum\;i}{n^{{}^{\circ}}\;of\;sampling\;point\;in\;stratum\;i}$$

(2) $$non-specificity=\frac{n^{{}^{\circ}}\;of\;sampling\;points\;assigned\;incorrectly\;to\;stratum\;i}{n^{{}^{\circ}}\;of\;sampling\;point\;do\;not\;belong\;to\;stratum\;i}$$

(3) $$predictive\;value=\frac{n^{{}^{\circ}}\;of\;sampling\;points\;assigned\;correctly\;to\;stratum\;i}{n^{{}^{\circ}}\;of\;sampling\;point\;that\;ROC\;method\;includes\;in\;stratum\;i}$$

After studying the functioning of both methods, the predictive capacity of each method was then analysed using the sound values of the sampling points of the other methods as controls [53,54]. The parameter used for this analysis was the prediction error (ε_*i*_), which is the difference between the measured value (control value) and the predicted value. The equations used to calculate the prediction error of the MTT road types (Equation (4)), and categorisation method (Equation (5)), respectively, were as follows:

(4) $$\varepsilon_{i}=P_{ij}-R_{i}$$

(5) $$\varepsilon_{i}=P_{ij}-C_{i}$$

The subscript “*i*” refers to the sampling point code (*P_i_*), road type code (*R_i_*) or road category code (*C_i_*), and the subscript “*j*” refers to the sampling methods in which the error is not being analysed. Next, the median prediction error obtained for each road category or road type was compared with the null value. For this, the Wilcoxon signed-rank test was applied [55]. This test determines whether the median of the prediction errors was biased. If the distribution of the prediction errors is unbiased, then a zero value will be obtained for the median.

Prediction errors of the different methods were also compared. To that end, the median absolute error of prediction (|ε_*i*_|) was analysed using the Mann-Whitney test [51]. If there is no significant difference it is assumed that the sampling methods have a similar predictive capacity.

Finally, the population exposed to noise was analysed and the population annoyed by noise was estimated. The demographic data of the geographic information system of the National Statistics Institute of Chile [56] were used to analyse the population exposed to noise. Noise levels registered in the road categories or road types were assigned to populations that reside in them [54]. Internationally validated equations were used to estimate the population annoyed by noise. Thus, the percentages of annoyed (%*A*) and highly annoyed (%*HA*) population were estimated from the *L_den_* descriptor with the following equations [57,58]:

(6) $$\%A=0.0001795{\left(L_{den}-37\right)}^{3}+0.0211{\left(L_{den}-37\right)}^{2}+0.5353\left(L_{den}-37\right)$$

(7) $$\%HA=0.0009868{\left(L_{den}-42\right)}^{3}-0.01436{\left(L_{den}-42\right)}^{2}+0.5118\left(L_{den}-42\right)$$

With respect to nocturnal noise, the percentages of population with little sleep disturbance (%*LSD*), sleep disturbance (%*SD*), and those who were highly sleep disturbed (%*HSD*) were estimated from L_n_ descriptor using the following equations [59]:

(8) $$\%LSD=\left(-8.4\right)+0.16L_{n}+0.01081{\left(L_{n}\right)}^{2}$$

(9) $$\%SD=13.8-0.85L_{n}+0.01670{\left(L_{n}\right)}^{2}$$

(10) $$\%HSD=20.8-1.05L_{n}+0.01486{\left(L_{n}\right)}^{2}$$

## 3. Results

### 3.1. Study of the Functioning of Sampling Methods

#### 3.1.1. Grid Method

Having calculated the sound values of *L_d_*, *L_n_* and *L_den_* descriptors in the different sampling points, the sound values of the different square grids were calculated. The results are shown in Table 1. Table 1 shows that the interquartile range of sound values registered in the cells is quite high. Previous studies [33,48] reported high uncertainties in the predictive capacity of the grid squares, due to the high variability of the sound levels among nearby streets with different functionality. Therefore, if the sound differences between adjacent sampling points are analysed, 69%, 49% and 59% are higher than 5 dB for *L_d_*, *L_n_* and *L_den_* descriptors, respectively.

#### 3.1.2. MTT Road Types

**Figure 3:**
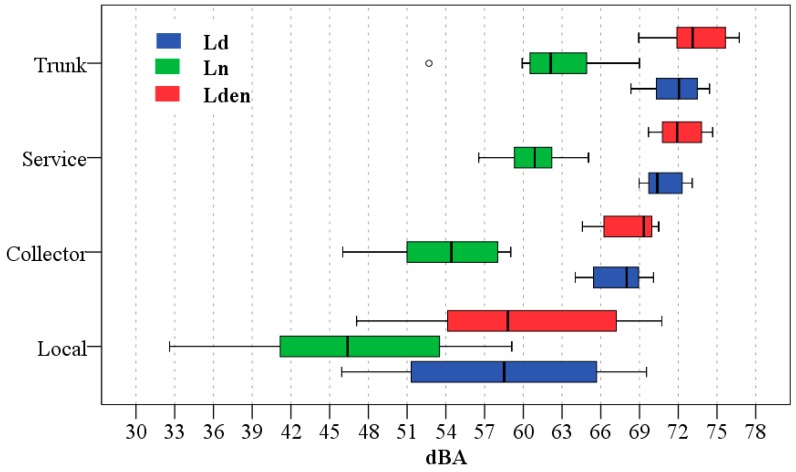
Box plot of *L_d_*, *L_n_* and *L_den_* descriptors registered in each road types.

This stratified sampling is based on the hypothesis that different strata—road types in this case—have significant differences in sound values. First, to resolve this hypothesis, a descriptive analysis through a box plot was carried out (Figure 3).

Figure 3 shows that average values of sound descriptors decrease from trunk to local road type. In highway road types, as previously indicated, only one sampling point was used. In this road type the sound values of 76.4 dB, 70.1 dB and 78.9 dB were registered for the *L_d_*, *L_n_* and *L_den_* descriptors, respectively. Figure 3 also shows the analysis of the variability in mean sound levels. Trunk and service road types have an overlap of interquartile range and local road types have a high variability.

The hypothesis was resolved first by using the Kruskal-Wallis test. This test indicated significant differences (*p*-value ≤ 0.001) for all the sound descriptors studied. Thus, the Mann-Whitney *U* test was then applied to analyse the differences among road type pairs (Table 2).

As shown in Table 2, the Mann-Whitney *U* test found no significant differences (*p*-value > 0.05) between trunk and service road types for *L_d_*, *L_n_* and *L_den_* descriptors. Nevertheless, for the remaining pairs of road types, significant differences (*p*-value ≤ 0.05) for all sound indicators analysed were found.

**Figure 4:**
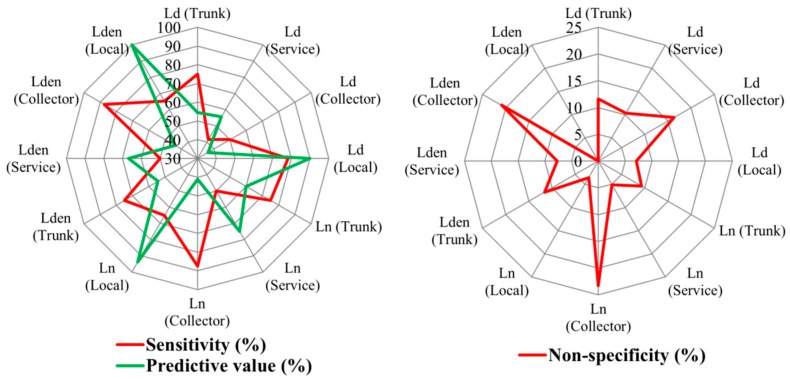
Results of *ROC* analysis for the different sound descriptors registered in the Ministry of Transport and Telecommunications road types.

In order to corroborate the quality of the previous results and to obtain more information about the MTT road types, the classification capacity of this method was then examined using *ROC* analysis. The results of this analysis are shown in Figure 4.

From the results shown in Figure 4, the following can be noted:

- Regarding the *ROC* sensitivity (%), which is a measure of the capacity to include previously assigned sampling points in the stratum, only the collector road type for *L_n_* and *L_den_* has values above 80%. The sensitivity has low percentages for the sound descriptors analysed, sometimes even lower than 50%, because of the presence of overlaps among trunk and service road types and the high variability of the local road type.
- Regarding the non-specificity (%), which measures the proportion of sampling points that were not initially assigned to a given stratum, but which the *ROC* analysis indicates belong to that stratum, only the local road type has values lower than 10% for all the sound descriptors. The collector road type also has high non-specificity values for all the sound descriptors, although it has high sensitivity values for *L_n_* and *L_den_*.
- Finally, with regard to the predictive values of the different road types (which represent the proportion of the sampling points that the *ROC* analysis assigned to the stratum that matched the road types to which they were initially assigned, relative to the total number of sampling points that the *ROC* analysis determined for the stratum) only the local road type has values above 80% for all the sound descriptors. The stratum predicted by the *ROC* analysis for local road types has a high percentage of sampling points that MTT had initially classified in this road type. However, other sampling points of local road types have high values and these points are classified in other road types according to *ROC* analysis. Therefore, the local road type has low sensitivity values.

#### 3.1.3. Categorisation Method

**Figure 5:**
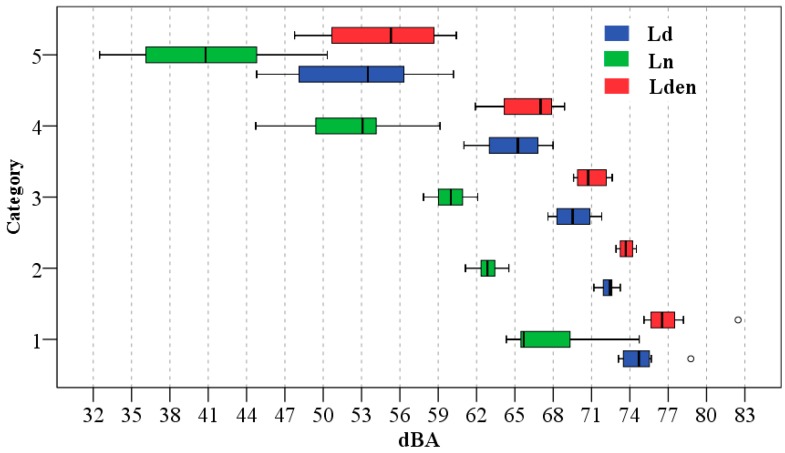
Box plot of *L_d_*, *L_n_* and *L_den_* descriptors registered in each road categories.

The different road categories defined by the method are based on the assumption of having significantly different noise levels. Therefore, like the MTT road types method, a descriptive and inferential analysis was conducted to test this hypothesis. The results of the descriptive analysis are shown in Figure 5.

In the box plot, the interquartile ranges of the different road categories and sound descriptors have no overlaps. Category 5 has the greatest variability but it is considerably lower than that presented by the local road type.

An inferential analysis was then conducted using the Kruskal-Wallis and Mann-Whitney tests. The Kruskal-Wallis test indicates significant differences (*p*-value ≤ 0.001) for all the sound descriptors studied. Thus, the Mann-Whitney *U* test with Holm correction was applied to analyse the differences among road category pairs (Table 3).

**Figure 6:**
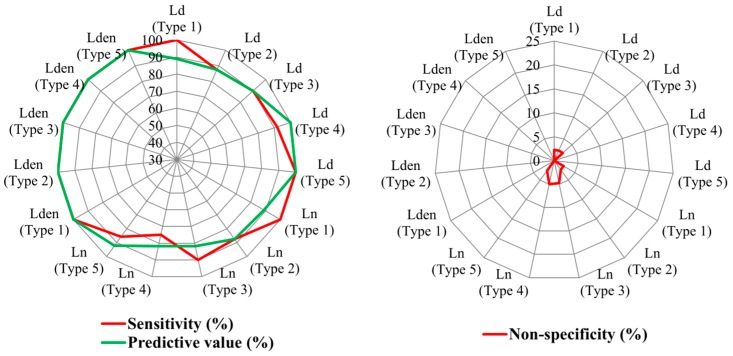
Results of *ROC* analysis for the different sound descriptors registered in the road categories.

As shown in Table 3, the Mann-Whitney *U* test found significant differences (*p*-value ≤ 0.01) among all pairs of road categories studied for all sound descriptors analysed. To corroborate the previous results, as carried out for the previous method, the classification capacity of the categorisation method was studied via *ROC* analysis. The results of this analysis are shown in Figure 6.

The results presented in Figure 6 show that the sensitivity of different sound descriptors is higher than 80% for all road categories (except the *L_n_* in Category 4), and even for the *L_den_* descriptor it is 100%. These high percentages are also obtained for the predictive value and therefore the percentages obtained in non-specificity are very low. They are lower than 5% in all sound descriptors.

These results differ from the previous method and it is therefore essential to compare the predictive capacity of both sampling methods. The results of this comparison are shown in the following section.

### 3.2. Predictive Capacity Analysis

In analysing the predictive capacity of the sampling methods, the sound values registered at the sampling points of the methods that were not being analysed were used.

To evaluate predictive capacity of the MTT road types, the sampling points chosen for the grid and categorisation method were used to compare the predictions of the MTT road types. All 104 sampling points evaluated in the grids and road categories could be associated with one of the road types (only one point was located in the highway road type, therefore, this road was not analysed). The sound values of these sampling points were compared with the mean value of the road type in which they were located and the prediction error was calculated using the difference between them (Equation (4)). The prediction error was analysed according to the road type where the control sampling point (*P_ij_*) was located. Table 4 shows the median from the error for the analysed sound descriptors.

Prediction errors of MTT road types are mostly lower than the 3 dB considered as suitable for estimations on noise maps. However, according to the Wilcoxon signed-rank test, errors by underestimation in trunk and service road types have significant differences with respect to the null value (except for the *L_den_* descriptor in the service road type). These two road types, as noted above, showed no significant difference in the average sound values registered. This fact directly affects the predictive capacity of the method.

The predictive capacity of the categorisation method was then analysed. To this end, using a similar procedure to that described above, the sampling points employed for the grid method and MTT road types were used to compare with the predictions of the road categories. All 104 of the sampling points evaluated in the grids and road types could be associated with one of the road categories. The sound values of these sampling points were compared with the mean value of the road category in which they were located and the prediction error was calculated using the difference between them (Equation (5)). The prediction error was analysed according to the road category where the control sampling point was located (*P_ij_*). Table 5 shows the median from the error for the sound descriptors analysed.

The prediction errors of the categorisation method are lower than 2 dB and have no significant differences with respect to the null value for all road categories and sound descriptors analysed (n.s.). These prediction errors are mostly lower compared with those of the MTT road types. However, to produce a detailed analysis of the differences in the estimation errors of the sampling methods, the median absolute errors of prediction were compared (|ε_*i*_|) using the Mann-Whitney test. The results are shown in Table 6.

To compare the predictive capacity of different sampling methods, the road type or road category where the control sampling point (*P_ij_*) was located was used as reference. Table 6 shows that the errors were higher for MTT road types for all sound descriptors analysed, regardless of road categories or road types taken as a reference. Taking the road category in which the control sampling point was placed as a reference, the error of L_n_ descriptor showed no significant differences between both sampling methods in Category 3 and 4. Taking the road type where the control sampling point was placed as a reference, the errors of both sampling methods in the collector road type showed no significant differences for all sound descriptors. The error of the night level in trunk and service road types and the error of the day, afternoon and night level in the trunk road type revealed no significant differences. Indeed, the differences in errors of both sampling methods are reduced if road types are taken as a reference. However, it is important to keep in mind that this classification had problems of statistical differentiation.

### 3.3. Calculation of Exposure Level and the Percentage of Annoyance

In the previous section the predictive capacity of sound values was analysed according to the different sampling methods. A sampling method that presents significant uncertainties of prediction will directly influence the calculation of the exposed population. Therefore, the variation in the level of exposed population and the percentage of annoyance depending on the sampling method used were analysed. In this study, the categorisation and MTT road type methods were analysed.

**Figure 7:**
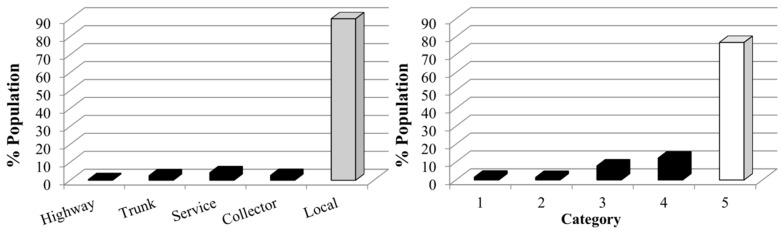
Population exposed to noise in different road categories and road types.

Figure 7 shows the percentage of exposed population according to the *L_den_* descriptors registered in different road categories and road types. Depending on the selected method, the results of population exposed to noise can change significantly. According to the MTT road types method, of the populations that reside in the highway, trunk, service and collector road type areas, 10% are exposed to levels higher than 65 dB. These areas whose *L_den_* > 65 dB are referred to as black acoustic zones [60]. However, in the case of the categorisation method, 23% of the population resides in black acoustic zones. Likewise, if the level of noise exposure in the road type and in the road category where a higher percentage of population resides is compared, the local road type population is in an acoustic grey zone (55 ≤ *L_den_* ≤ 65), whereas in Category 5 the population is in a white acoustic zone (*L_den_* < 55). Therefore, the differences in the capacity of sound prediction can clearly be misleading in the calculation of the percentage of exposed population.

**Figure 8:**
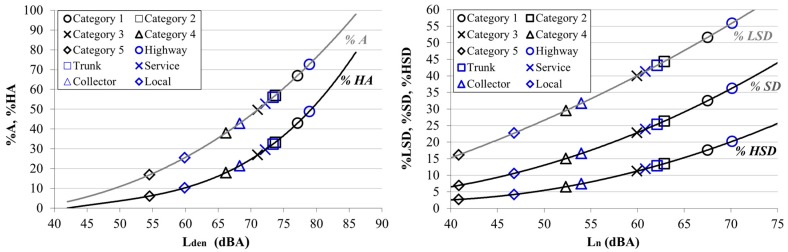
Percentages annoyance indicators (percentages of annoyed (%*A*) and highly annoyed (%*HA*) population; percentages of lowly sleep disturbed (%*LSD*), sleep disturbed (%*SD*) and highly sleep disturbed (%*HSD*) population) obtained from the proposed equations [58,59] for road types and road categories.

Finally, we calculated the percentages of annoyed population and percentages of the population who are sleep disturbed by noise using both the MTT road types and the categorisation method. The results are shown in Figure 8.

The results show that different road types have percentages of annoyance and sleep disturbed by noise higher than those registered in the different road categories. Those road types that register higher noise levels, and therefore higher levels of noise annoyance, are those that had a higher level of sound prediction uncertainty. The trunk and service road type have similar percentages of annoyance to Categories 2 and 3. However, in previous analysis significant problems of differentiation between these two road types were found. Furthermore, the difference in the percentages of annoyance between the local road type and Category 5 should be noted, being those with lower noise levels. These differences were also detected in the analysis of sound exposure.

## 4. Discussion

The variability of sound values registered in the grid squares of Talca is quite high. This result indicates a low predictive capacity of the grid method to assess the noise exposure. If the interquartile range obtained in the cells is compared with that obtained in the local road type and in Category 5 (the road type and road category with the highest variability of noise levels), more than 50% and 75% of the grids register a greater value, respectively. Indeed, the grid size is quite high; however, as stated above, in this study has been considered relevant to use the same number of sampling points in each measurement method. Following the instructions of the ISO 1996-2 [42], if intermediate grid points would be added when the sound differences between adjacent grid points were higher than 5 dB, a new sampling would have carried out with a number of similar points. However, as shown in previous studies [33], the selection of new sampling points does not guarantee a difference between adjacent points lower than 5 dB. Consequently, this method was not used in order to compare the uncertainties between different sampling methods.

Regarding the MTT road types, the results show an overlap of interquartile range of the sound values registered in the trunk and service road types. Also, the local road type has a high sound variability. These results are similar to those obtained in other studies carried out in cities of Chile with legislative road classification [38]. Consequently, the *ROC* analysis indicates that this method has a low percentage of sensitivity and predictive capacity and a high percentage of non-specificity. Nevertheless, the sound values in the different road categories of the categorisation method have highly significant statistical differences. The road categories also have a high percentage of sensitivity and predictive capacity and a very low percentage of specificity. 

The prediction errors of the categorisation method are lower than those of the MTT method for the different urban roads analysed. These differences in the prediction of sound values involve differences in the estimation of exposure levels and percentage of annoyance. According to the MTT method, 10% of the population is exposed to *L_den_* > 65 dB, whereas this is 23% of population according to the categorisation method. Also, as shown in Figure 8, road types have percentages of annoyance and sleep disturbed by noise higher than those registered by road categories.

Finally, the exposed population and the percentage of annoyance obtained using the categorisation method were compared with the results obtained in other cities. Lee *et al.* [28] carried out a recent acoustic study in Seoul (S, Korea) and the percentage obtained from population that exceeds the level of 65 dB for the *L_d_* descriptor and the level of 55 dB for *L_n_* descriptor were compared with European cities. In Talca 11% of the population (Category 1, 2 and 3) is exposed to average levels at daytime that are higher than 65 dB and to average levels at night that are higher than 55 dB. For both time periods these percentages are higher than those obtained in the cities of Helsinki (Finland) and Berlin (Germany), and are similar to those obtained in cities such as Frankfurt (Germany). However, these percentages are lower than those obtained the cities of Seoul, Copenhagen (Denmark) and Madrid (Spain). In a further acoustic study recently carried out by Braubach *et al.* [15] in the cities of Basel (Switzerland), Rotterdam (The Netherlands) and Thessaloniki (Greece), limit values of 64 dB (annoyance by noise), 67.5 dB (major noise problem), and 65 dB (major noise problem) were found using the *L_den_* descriptor. The population of Talca residing in Category 1 to Category 4 is exposed to levels greater than 64 and 65 dB for the *L_den_* descriptor and for Category 1 to Category 3 the population is exposed to levels higher than 65.5 dB. Therefore, 23% and 14% of the population is exposed to values greater than 64–65 dB and 67.5 dB respectively. These percentages are much higher than those obtained in the cities of Basel, Rotterdam and Thessaloniki.

## 5. Conclusions

The selection of a suitable sampling method is essential to achieve an accurate assessment of the impact of noise pollution on the population. The grid, MTT road types and categorisation methods were analysed in the city of Talca (Chile). The major conclusions drawn from the results are as follows:

The grid squares have a high variability of sound values. This high variability leads to differences in sound values registered at adjacent points of more than 5 dB in 69%, 49% and 59% for *L_d_*, *L_n_* and *L_den_* descriptors, respectively. 

The MTT road types have a low percentage of sensitivity and predictive capacity (except for the collector road type for *L_n_* and *L_den_* that has values above 80% of sensitivity and for the local road type for all the sound descriptors that has values above 80% of predictive capacity) and a high percentage of non-specificity (except for the local road type for all the sound descriptors that has values lower than 10%). This low discrimination and predictive capacity is caused, among other factors, by the lack of significant differentiation of sound values registered in trunk and service road types and by the high variability of the sound values of the local road type.

Average sound values in the different road categories of the categorisation method have highly significant statistical differences. The road categories also have a high percentage of sensitivity (>75%) and predictive capacity (>80%) and a very low percentage of specificity (<5%). Therefore, the functional stratification of noise levels observed in European cities that were studied previously is also found in Chilean cities. These results suggest a great advance in the validity of the categorisation method because of its application in a Chilean city.

The predictive capacity of the categorisation method is higher than that of the MTT method. This difference in the predictive capacity of sound values involves differences in the estimation of exposure levels and in the percentage of annoyance. Consequently, the categorisation method is more accurate than the MTT method to assess the impact of noise pollution on the population.

Talca is a city affected by noise pollution and also by its related problems of public health of its inhabitants. The percentages of population exposed to daytime and nighttime sound levels that are harmful to health are higher than those obtained in Helsinki and Berlin. Furthermore, the percentage of exposed population to *L_den_* > 64 dB is much higher than that obtained in the cities of Basel, Rotterdam and Thessaloniki.

## Figures and Tables

**Table 1:** Median (M_e_) and interquartile range (IQR) of *L_d_*, *L_n_* and *L_den_* descriptors registered in the square grids.

Grid	*L_d_* (dBA)		*L_n_* (dBA)		*L_den_* (dBA)		Grid	*L_d_* (dBA)		*L_n_* (dBA)		*L_den_* (dBA)	
	M_e_	IQR	M_e_	IQR	M_e_	IQR		M_e_	IQR	M_e_	IQR	M_e_	IQR
**1**	51.4	12.8	44.0	16.8	53.1	13.9	**19**	62.7	21.8	54.9	23.0	63.6	17.8
**2**	59.4	17.8	53.0	21.7	63.0	18.2	**20**	51.7	11.9	44.5	10.7	55.3	5.6
**3**	64.9	9.3	53.1	17.9	65.5	10.2	**21**	64.7	14.4	52.3	23.3	65.7	15.3
**4**	64.9	14.6	50.3	24.7	65.5	15.9	**22**	72.3	5.8	62.2	7.3	73.8	5.4
**5**	53.2	16.6	41.8	21.1	53.9	16.4	**23**	67.2	17.2	59.0	19.9	69.1	16.8
**6**	48.7	4.7	43.4	3.3	52.4	3.4	**24**	62.2	13.9	52.8	20.0	63.8	14.4
**7**	62.5	22.2	52.9	24.1	63.2	16.9	**25**	55.2	22.0	49.2	12.9	58.9	17.6
**8**	69.0	20.0	59.0	10.3	70.3	14.2	**26**	51.8	11.3	43.8	6.5	55.3	6.2
**9**	61.0	21.5	50.6	20.1	62.1	21.1	**27**	64.7	15.4	51.5	11.0	65.7	14.0
**10**	50.3	5.4	42.3	3.7	52.5	3.8	**28**	69.7	8.5	58.4	11.2	70.5	8.6
**11**	56.7	16.4	47.7	15.1	58.6	15.6	**29**	66.4	19.0	51.5	26.1	67.1	19.5
**12**	57.3	12.0	51.5	19.3	60.8	11.9	**30**	61.4	8.1	45.2	17.0	61.8	9.4
**13**	55.1	11.3	47.0	12.1	57.9	7.5	**31**	60.7	23.9	47.9	23.6	61.9	22.9
**14**	55.3	10.1	35.8	10.5	55.8	4.0	**32**	57.5	22.3	41.7	19.3	58.4	20.6
**15**	62.2	24.6	53.8	25.3	63.3	19.3	**33**	60.1	12.6	47.1	16.0	61.4	12.5
**16**	71.6	16.3	61.0	18.7	73.1	16.5	**34**	62.1	21.3	50.3	21.4	63.4	20.4
**17**	58.4	17.8	49.6	19.7	60.1	18.4	**35**	51.0	18.6	41.6	14.5	53.3	17.4
**18**	65.7	14.9	58.6	15.7	67.9	14.9							

**Table 2:** *p*-Values with Holm adjustment of pairwise comparisons of road types using Mann-Whitney *U* test.

***L_d_***	**Road Types**	**Trunk**	**Service**	**Collector**
	**Service**	0.343	-	-
	**Collector**	0.005	0.003	-
	**Local**	<0.001	<0.001	0.005
***L_n_***	**Road types**	**Trunk**	**Service**	**Collector**
	**Service**	0.208	-	-
	**Collector**	0.009	0.002	-
	**Local**	<0.001	<0.001	0.046
***L_den_***	**Road types**	**Trunk**	**Service**	**Collector**
	**Service**	0.270	-	-
	**Collector**	0.008	<0.001	-
	**Local**	<0.001	<0.001	0.008

**Table 3:** *p*-Values with Holm adjustment of pairwise comparisons of road categories using Mann-Whitney *U* test.

***L_d_***	**Category**	**1**	**2**	**3**	**4**
	**2**	<0.001	-	-	-
	**3**	<0.001	<0.001	-	-
	**4**	<0.001	<0.001	<0.001	-
	**5**	<0.001	<0.001	<0.001	<0.001
***L_n_***	**Category**	**1**	**2**	**3**	**4**
	**2**	0.002	-	-	-
	**3**	0.001	0.002	-	-
	**4**	0.001	0.001	0.001	-
	**5**	0.001	<0.001	<0.001	<0.001
***L_den_***	**Category**	**1**	**2**	**3**	**4**
	**2**	<0.001	-	-	-
	**3**	<0.001	<0.001	-	-
	**4**	<0.001	<0.001	<0.001	-
	**5**	<0.001	<0.001	<0.001	<0.001

**Table 4:** Prediction errors (ε) of Ministry of Transport and Telecommunications road types for *L_d_*, *L_n_* and *L_den_* descriptors.

Road Types	No. Points	ε_*Ld*_	ε_*Ln*_	ε_*Lden*_
Trunk	13	1.3 *	2.2 **	2.3 ^n.s.^
Service	13	1.3 *	1.3 *	1.5 *
Collector	3	0.5 ^n.s.^	4.1 ^n.s.^	0.8 ^n.s.^
Local	74	−1.0 ^n.s.^	−0.3 ^n.s.^	−0.6 ^n.s.^

No.: Number; * Significant at *p* ≤ 0.05; ** Significant at *p* ≤ 0.01; ^n.s.^ Non-significant difference (*p* > 0.05).

**Table 5:** Prediction errors (ε) of the categorisation method for *L_d_*, *L_n_* and *L_den_* descriptors.

Category	No. Points	ε_*Ld*_	ε_*Ln*_	ε_*Lden*_
1	5	−0.5 ^n.s.^	−2.0 ^n.s.^	−0.8 ^n.s.^
2	11	0.2 ^n.s.^	−0.4 ^n.s.^	−0.1 ^n.s.^
3	21	−0.1 ^n.s.^	0.0 ^n.s.^	0.2 ^n.s.^
4	21	1.1 ^n.s.^	0.7 ^n.s.^	0.7 ^n.s.^
5	46	−1.0 ^n.s.^	1.2 ^n.s.^	0.2 ^n.s.^

No.: Number; ^n.s.^ Non-significant difference (*p* > 0.05).

**Table 6:** Absolute values of prediction errors (|ε|) for *L_d_*, *L_n_* and *L_den_* for road types and road categories and comparison to prediction errors of both methods (Categorisation and Ministry of Transport and Telecommunication (MTT)) using Mann-Whitney *U* test.

Reference Road	Method	No. Points	|ε|_*Ld*_	Sig.	|ε|_*Ln*_	Sig.	|ε|_*Lden*_	Sig.
**Category 1**	Categorisation	5	1.1	**	2.0	**	1.5	**
	MTT	8	2.9		3.5		3.0	
**Category 2**	Categorisation	11	0.2	**	0.7	*	0.3	*
	MTT	13	1.3		1.3		1.5	
**Category 3**	Categorisation	21	0.6	**	1.0	^n.s.^	1.0	*
	MTT	15	9.7		11.1		9.7	
**Category 4**	Categorisation	21	2.0	***	3.9	^n.s.^	1.6	***
	MTT	21	5.1		5.5		6.5	
**Category 5**	Categorisation	46	4.2	*	3.5	*	2.7	**
	MTT	46	5.1		5.6		5.0	
**Trunk**	MTT	13	2.0	*	2.2	^n.s.^	2.3	^n.s.^
	Categorisation	13	1.1		1.5		0.8	
**Service**	MTT	13	1.6	*	1.3	^n.s.^	1.8	*
	Categorisation	17	0.5		0.9		0.5	
**Collector**	MTT	3	0.8	^n.s.^	4.1	^n.s.^	1.6	^n.s.^
	Categorisation	10	0.9		5.2		1.3	
**Local**	MTT	74	6.6	***	6.5	***	6.5	***
	Categorisation	64	2.9		3.5		2.0	

No.: Number; Sig.: Significance; * Significant at *p* ≤ 0.05; ** Significant at *p* ≤ 0.01; *** Significant at *p* ≤ 0.001; ^n.s.^ Non-significant difference (*p* > 0.05).
